# Crystal structure and Hirshfeld surface analysis of 3,6-bis­(pyrimidin-2-yl)-1,4-di­hydro-1,2,4,5-tetra­zine dihydrate

**DOI:** 10.1107/S2056989020002765

**Published:** 2020-03-03

**Authors:** Kenika Khotchasanthong, Siripak Jittirattanakun, Kittipong Chainok

**Affiliations:** a Multifunctional Crystalline Materials and Applications, Division of Chemistry, Faculty of Science and Technology, Thammasat University, Khlong Luang, Pathum Thani, 12121, Thailand; b Multifunctional Crystalline Materials and Applications, Materials and Textile Technology, Faculty of Science and Technology, Thammasat University, Khlong Luang, Pathum Thani, 12121, Thailand

**Keywords:** crystal structure, hydrogen bonds, Hirshfeld surface, pyrimidine

## Abstract

In the title compound, C_10_H_8_N_8_·2H_2_O or H_2_bmtz·2H_2_O [bmtz = 3,6-bis­(2′-pyrimid­yl)-1,2,4,5-tetra­zine], the asymmetric unit consists of one-half mol­ecule of H_2_bmtz and one water mol­ecule, the whole H_2_bmtz mol­ecule being generated by a crystallographic twofold rotation axis passing through the middle point of the 1,4-di­hydro-1,2,4,5-tetra­zine moiety. In the crystal, N—H⋯O, N—H⋯N, O—H⋯O hydrogen bonds and aromatic π–π stacking inter­actions link the components into a three-dimensional supra­molecular network. Hirshfeld surface analysis was used to further investigate the inter­molecular inter­actions in the crystal structure.

## Abstract 2

In the crystal of the title compound, the packing is driven by O—H⋯O, O—H⋯N and N—H⋯N hydrogen-bond inter­actions along with π–π stacking inter­actions.

## Chemical context   

The chemistry of nitro­gen-containing heterocyclic compounds has attracted the attention of the scientific community for over a century. Many compounds of this class are bioactive (Jubeen *et al.*, 2018 ▸) and show promising pharmacological properties (Alcaide *et al.*, 2016 ▸; Varano *et al.*, 2016 ▸). Among these, numerous pyrimidine derivatives have been studied extensively in the context of synthetic organic chemistry and coordination chemistry (Kaim, 2002 ▸). For instance, the tetra­zine-based ligand 3,6-bis­(2′-pyrimid­yl)-1,2,4,5-tetra­zine (bmtz) has been used as a polydentate ligand for the formation of silver(I) coordination polymers (Chainok *et al.*, 2012 ▸) and for the self-assembly of the highly stable Fe^II^ penta­gonal metallacycles (Giles *et al.*, 2011 ▸). Herein, the crystal and mol­ecular structures of the di­hydro­tetra­zine-based compound 3,6-bis(pyrimidin-2-yl)-1,4-di­hydro-1,2,4,5-tetra­zine dihydrate, C_10_H_8_N_8_·2H_2_O or H_2_bmtz·2H_2_O (**I**), is described along with an analysis of its Hirshfeld surface.
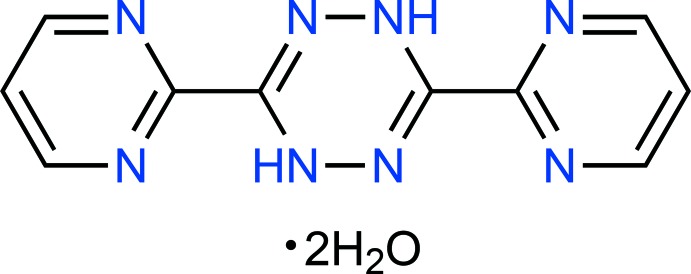


## Structural commentary   

**Figure 1:**
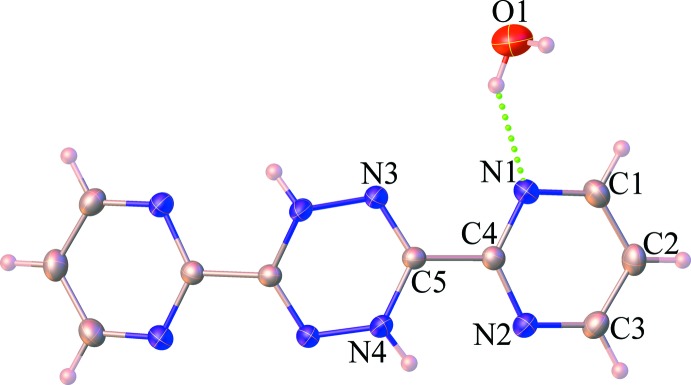
Mol­ecular structure of (**I**) with displacement ellipsoids drawn at the 50% probability level. Unlabelled atoms are generated by the symmetry operation −*x*, *y*, 3/2 − z.

The mol­ecular structure of (**I**) is shown in Fig. 1 ▸. The asymmetric unit consists of one-half mol­ecule of H_2_bmtz and one water mol­ecule, in which the whole mol­ecule of the H_2_bmtz is generated by a crystallographic twofold rotation axis passing through the middle point of the 1,4-di­hydro-1,2,4,5-tetra­zine moiety. The H_2_bmtz mol­ecule is therefore not planar (r.m.s. deviation from planarity = 0.598 Å) with a C4—C5—N3—N4^i^ torsion angle of 178.46 (14)° [symmetry code: (i) −*x*, *y*, 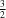 − *z*]. The pyrimidine rings are twisted with respect to each other, making a dihedral angle of 43.67 (9)°. The 1,4-di­hydro-1,2,4,5-tetra­zine moiety adopts a twist-boat conformation with a C5—N3—N4^i^—C5^i^ torsion angle of −41.17 (17)°. The N3—N4^i^ and C5—N4 bond lengths of 1.423 (2) and 1.395 (2) Å, confirm their single-bond character, while the C3—N5 bond length of 1.278 (2) Å, is consistent with a double bond (compare QORNAM, Glöckle *et al.*, 2001 ▸; ZASTAQ, Chainok *et al.*, 2012 ▸). The C—C and C—N bond lengths in the pyrimidine ring are characteristic for a delocalized double bond and a typical single bond (QORNAM, Glöckle *et al.*, 2001 ▸).

## Supra­molecular features   

**Figure 2:**
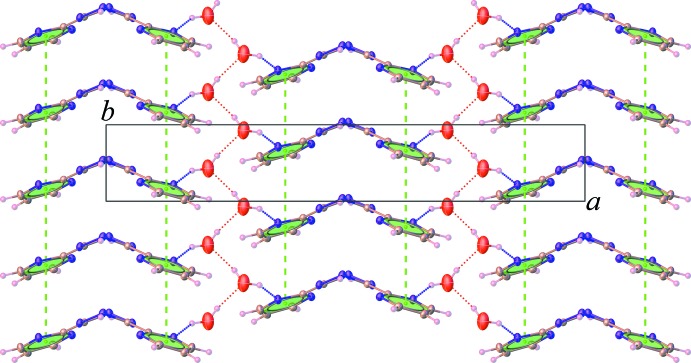
Partial packing diagram of (**I**), showing the O—H⋯O and O—H⋯N hydrogen bonds (dashed lines) and π–π stacking inter­actions propagating in the *ab* plane.

**Figure 3:**
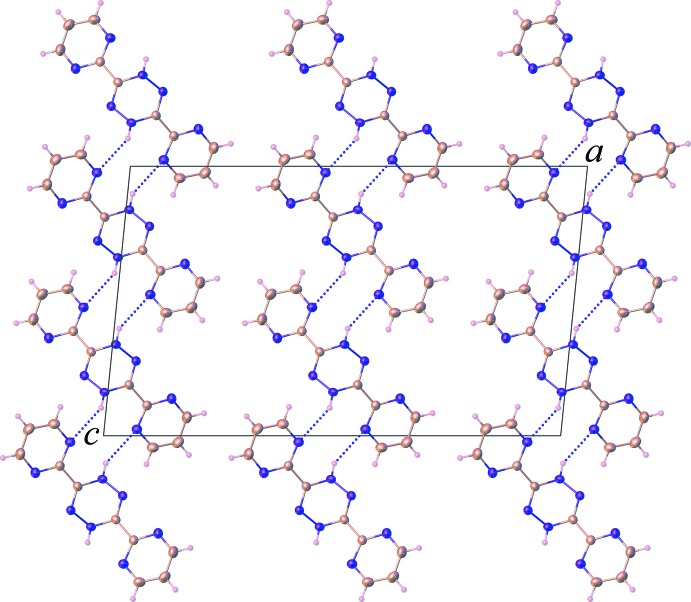
Partial packing diagram of (**I**) viewed along the *b* axis, showing the N—H⋯N hydrogen bonds (dashed lines).

In the crystal, the H_2_bmtz mol­ecules are stacked along [010] into columns through π–π inter­actions between the pyrimidine rings [centroid-to-centroid distance = 3.726 (2) Å]. At the same time, the water mol­ecules are connected by O—H⋯O hydrogen bonds (Table 1 ▸), resulting in the formation of a zigzag chain. These motifs are then connected together through N—H⋯O hydrogen bonds involving the tetra­zine nitro­gen atoms and the water mol­ecules to form a sheet structure propagating in the *ab* plane, as shown in Fig. 2 ▸. The sheets are further linked into an overall three-dimensional supra­molecular network through N—H⋯N hydrogen bonds with an 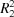
(10) ring motif, Fig. 3 ▸, which involve the di­hydro nitro­gen atoms and the pyrimidine nitro­gen atoms. A weak C—H⋯O inter­action is also noted (Table 1 ▸).

## Hirshfeld surface analysis   

**Figure 4:**
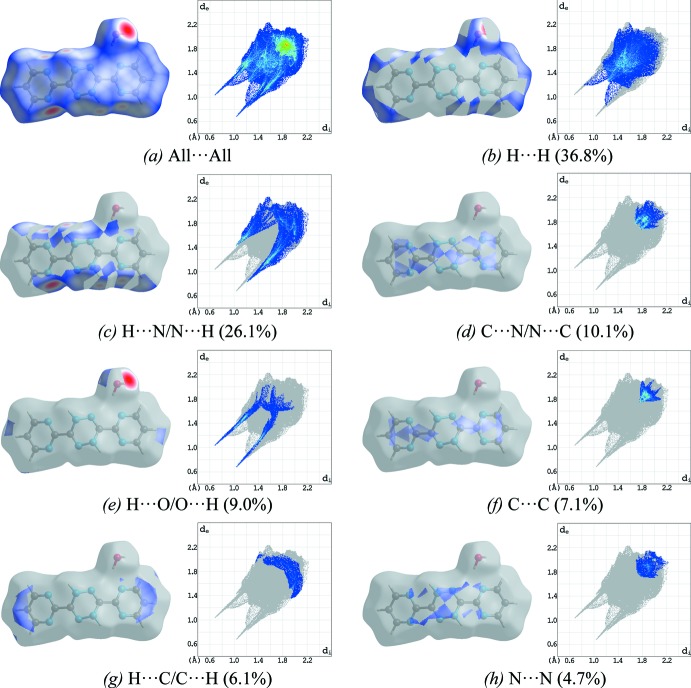
Two-dimensional fingerprint plots of the title compound (**I**), showing (*a*) all inter­actions, and those delineated into (*b*) H⋯H, (*c*) H⋯N/N⋯H, (*d*) C⋯N/N⋯C, (*e*) H⋯O/O⋯H, (*f*) C⋯C, (*g*) H⋯C/C⋯H, and (*h*) N⋯N contacts [*d*_e_ and *d*_i_ represent the distances from a point on the Hirshfeld surface to the nearest atoms outside (external) and inside (inter­nal) the surface, respectively].

To further qu­antify the nature of the inter­molecular inter­actions present in the crystal structure, Hirshfeld surfaces (McKinnon *et al.*, 2007 ▸) and their associated two-dimensional fingerprint plots (Spackman & McKinnon, 2002 ▸) were generated using *CrystalExplorer17* (Turner *et al.*, 2017 ▸). The shorter and longer contacts are indicated as red and blue spots, respectively, on the Hirshfeld surfaces, and contacts with distances approximately equal to the sum of the van der Waals radii are represented as white spots. The contribution of inter­atomic contacts to the *d*_norm_ surface of the title compound is shown in Fig. 4 ▸. Analysis of the two-dimensional fingerprint plots, Fig. 4 ▸, reveals that H⋯H (36.8%) contacts are the major contributors toward the Hirshfeld surface, whereas H⋯N/N⋯H (26.1%) contacts (*i.e.* N—H⋯N) make a less significant contribution. The contribution of the H⋯O/O⋯H (9.0%) contacts (*i.e.* C—H⋯O and O—H⋯O) and other contacts such as C⋯C (7.1%) (*i.e.* π–π stacking), H⋯C/C⋯H (6.1%) and N⋯N (4.7%) make a small contribution to the entire Hirshfeld surface.

## Database survey   

A search of the Cambridge Crystallographic Database (CSD version 5.41, November 2019 update; Groom *et al.*, 2016 ▸) using *ConQuest* gave 4261 hits, reflecting the large number of pyrimidine-containing heterocyclic compounds that have been characterized. However, searches for compounds related to H_2_bmtz yielded just two hits for *μ*_2_-1,4-di­hydro-3,6-bis­[(2′-pyrimid­yl)-1,2,4,5-tetra­zine]bis­[bis­(tri­phenyl­phosphine)cop­per(I)] bis­(tetra­fluorido­borate) di­chloro­methane solvate (QORNAM, Glöckle *et al.*, 2001 ▸) and *catena*-[[*μ*_2_-3,6-di(pyrimidin-2-yl)-1,4-di­hydro-1,2,4,5-tetra­zine][*μ*_2_-(di­cyano­ethen­yl­­idene)amido][(di­cyano­ethenyl­idene)amido]­aceto­nitrile­disilver(I)] (ZASTAQ, Chainok *et al.*, 2012 ▸).

## Synthesis and crystallization   

All commercially available chemicals and solvents were of reagent grade and were used as received without further purification. H_2_bmtz was synthesized according to a literature method (Kaim & Fees, 1995 ▸). Single crystals for X-ray structure analysis were obtained by recrystallization from mixed solvents of CH_2_Cl_2_/H_2_O (1:1, *v*/*v*).

## Refinement   

Crystal data, data collection and structure refinement details are summarized in Table 2 ▸. All H atoms were located in difference-Fourier maps: the carbon-bound H atoms were relocated to idealized positions and refined as riding atoms with C—H = 0.93 Å and *U*_iso_(H) = 1.2*U*_eq_(C). The 1,4-di­hydro-1,2,4,5-tetra­zine and water H atoms were located in difference-Fourier maps and were constrained to N—H = 0.86 ± 0.01 Å with *U*_iso_(H) = 1.2*U*_eq_(N) and O—H = 0.84 ± 0.01 Å with *U*_iso_(H) = 1.5*U*_eq_(O), respectively.

## Supplementary Material

Crystal structure: contains datablock(s) I, global. DOI: 10.1107/S2056989020002765/hb7895sup1.cif

Structure factors: contains datablock(s) I. DOI: 10.1107/S2056989020002765/hb7895Isup2.hkl

Supporting information file. DOI: 10.1107/S2056989020002765/hb7895Isup3.cdx

Supporting information file. DOI: 10.1107/S2056989020002765/hb7895Isup4.cml

CCDC reference: 1986751

Additional supporting information:  crystallographic information; 3D view; checkCIF report

## Figures and Tables

**Table 1:** Hydrogen-bond geometry (Å, °)

*D*—H⋯*A*	*D*—H	H⋯*A*	*D*⋯*A*	*D*—H⋯*A*
O1—H1*A*⋯O1^i^	0.88 (1)	1.81 (1)	2.642 (4)	156 (2)
O1—H1*B*⋯N1	0.86 (1)	2.15 (4)	2.863 (3)	140 (5)
N4—H4⋯N2^ii^	0.85 (1)	2.57 (1)	3.221 (1)	133 (2)
C2—H2⋯O1^iii^	0.93	2.43	3.278 (3)	151

Symmetry codes: (i) 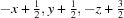; (ii) 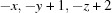; (iii) 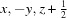.

**Table 2:** Experimental details

Crystal data	
Chemical formula	C_10_H_8_N_8_·2H_2_O
*M*_r_	276.28
Crystal system, space group	Monoclinic, *C*2/*c*
Temperature (K)	296
*a*, *b*, *c* (Å)	23.4730 (12), 3.7262 (2), 13.9102 (7)
β (°)	95.687 (2)
*V* (Å^3^)	1210.67 (11)
*Z*	4
Radiation type	Mo *K*α
μ (mm^−1^)	0.11
Crystal size (mm)	0.32 × 0.2 × 0.2

Data collection	
Diffractometer	Bruker D8 QUEST CMOS PHOTON II
Absorption correction	Multi-scan (*SADABS*; Bruker, 2016 ▸)
*T*_min_, *T*_max_	0.714, 0.746
No. of measured, independent and observed [*I* > 2σ(*I*)] reflections	11339, 1487, 1145
*R*_int_	0.030
(sin θ/λ)_max_ (Å^−1^)	0.667

Refinement	
*R*[*F* ^2^ > 2σ(*F* ^2^)], *wR*(*F* ^2^), *S*	0.053, 0.174, 1.05
No. of reflections	1487
No. of parameters	103
No. of restraints	4
H-atom treatment	H atoms treated by a mixture of independent and constrained refinement
Δρ_max_, Δρ_min_ (e Å^−3^)	0.26, −0.39

Computer programs: *APEX3* and *SAINT* (Bruker, 2016 ▸), *SHELXT* (Sheldrick, 2015*a* ▸), *SHELXL2018* (Sheldrick, 2015*b* ▸) and *OLEX2* (Dolomanov *et al.*, 2009 ▸).

## supplementary crystallographic information

### Crystal data

**Table:**

C_10_H_8_N_8_·2H_2_O	*F*(000) = 576
*M**_r_* = 276.28	*D*_x_ = 1.516 Mg m^−^^3^
Monoclinic, *C*2/*c*	Mo *K*α radiation, λ = 0.71073 Å
*a* = 23.4730 (12) Å	Cell parameters from 4681 reflections
*b* = 3.7262 (2) Å	θ = 3.3–28.1°
*c* = 13.9102 (7) Å	µ = 0.11 mm^−^^1^
β = 95.687 (2)°	*T* = 296 K
*V* = 1210.67 (11) Å^3^	Block, orange
*Z* = 4	0.32 × 0.2 × 0.2 mm

### Data collection

**Table:**

Bruker D8 QUEST CMOS PHOTON II diffractometer	1487 independent reflections
Radiation source: sealed x-ray tube	1145 reflections with *I* > 2σ(*I*)
Graphite monochromator	*R*_int_ = 0.030
Detector resolution: 7.39 pixels mm^-1^	θ_max_ = 28.3°, θ_min_ = 2.9°
φ and ω scans	*h* = −30→29
Absorption correction: multi-scan (SADABS; Bruker, 2016)	*k* = −4→4
*T*_min_ = 0.714, *T*_max_ = 0.746	*l* = −18→18
11339 measured reflections	

### Refinement

**Table:**

Refinement on *F*^2^	Primary atom site location: dual
Least-squares matrix: full	Hydrogen site location: mixed
*R*[*F*^2^ > 2σ(*F*^2^)] = 0.053	H atoms treated by a mixture of independent and constrained refinement
*wR*(*F*^2^) = 0.174	*w* = 1/[σ^2^(*F*_o_^2^) + (0.1061*P*)^2^ + 0.7279*P*] where *P* = (*F*_o_^2^ + 2*F*_c_^2^)/3
*S* = 1.05	(Δ/σ)_max_ < 0.001
1487 reflections	Δρ_max_ = 0.26 e Å^−^^3^
103 parameters	Δρ_min_ = −0.39 e Å^−^^3^
4 restraints	

### Special details

**Table:**

Geometry. All esds (except the esd in the dihedral angle between two l.s. planes) are estimated using the full covariance matrix. The cell esds are taken into account individually in the estimation of esds in distances, angles and torsion angles; correlations between esds in cell parameters are only used when they are defined by crystal symmetry. An approximate (isotropic) treatment of cell esds is used for estimating esds involving l.s. planes.

### Fractional atomic coordinates and isotropic or equivalent isotropic displacement parameters (Å^2^)

**Table:**

	*x*	*y*	*z*	*U*_iso_*/*U*_eq_	
O1	0.21290 (11)	0.4286 (11)	0.71824 (16)	0.1166 (10)	
H1A	0.2318 (9)	0.594 (5)	0.7537 (16)	0.060 (8)*	
H1B	0.1796 (10)	0.400 (13)	0.738 (3)	0.19 (2)*	
N1	0.14093 (6)	0.2128 (5)	0.86243 (11)	0.0426 (4)	
N2	0.07173 (6)	0.2495 (4)	0.97609 (10)	0.0386 (4)	
N3	0.05508 (6)	0.4046 (4)	0.72282 (9)	0.0372 (4)	
N4	−0.00726 (6)	0.5326 (4)	0.83844 (9)	0.0381 (4)	
H4	−0.0111 (9)	0.481 (6)	0.8970 (8)	0.052 (6)*	
C1	0.17826 (8)	0.0798 (6)	0.93187 (14)	0.0488 (5)	
H1	0.215005	0.024291	0.916985	0.059*	
C2	0.16473 (8)	0.0213 (5)	1.02458 (14)	0.0468 (5)	
H2	0.190969	−0.077223	1.071768	0.056*	
C3	0.11048 (8)	0.1160 (5)	1.04382 (13)	0.0441 (5)	
H3	0.100261	0.086189	1.106236	0.053*	
C4	0.08877 (6)	0.2840 (4)	0.88786 (11)	0.0327 (4)	
C5	0.04524 (7)	0.4139 (4)	0.81150 (11)	0.0323 (4)	

### Atomic displacement parameters (Å^2^)

**Table:**

	*U*^11^	*U*^22^	*U*^33^	*U*^12^	*U*^13^	*U*^23^
O1	0.0846 (16)	0.196 (3)	0.0734 (14)	−0.0089 (18)	0.0266 (13)	−0.0020 (18)
N1	0.0327 (8)	0.0553 (9)	0.0399 (8)	0.0017 (6)	0.0047 (6)	−0.0006 (7)
N2	0.0387 (8)	0.0453 (8)	0.0319 (7)	0.0027 (6)	0.0038 (6)	0.0021 (6)
N3	0.0305 (7)	0.0496 (9)	0.0316 (7)	−0.0050 (6)	0.0040 (5)	−0.0019 (6)
N4	0.0353 (7)	0.0527 (9)	0.0263 (7)	0.0066 (6)	0.0039 (5)	−0.0024 (6)
C1	0.0348 (9)	0.0596 (12)	0.0516 (11)	0.0081 (8)	0.0020 (8)	−0.0018 (9)
C2	0.0453 (10)	0.0468 (10)	0.0460 (10)	0.0080 (8)	−0.0072 (8)	0.0021 (8)
C3	0.0503 (11)	0.0476 (10)	0.0342 (8)	0.0041 (8)	0.0034 (7)	0.0033 (7)
C4	0.0316 (8)	0.0331 (8)	0.0334 (8)	−0.0012 (6)	0.0029 (6)	−0.0029 (6)
C5	0.0313 (8)	0.0347 (8)	0.0312 (8)	−0.0022 (6)	0.0054 (6)	−0.0014 (6)

### Geometric parameters (Å, º)

**Table:**

O1—H1A	0.882 (10)	N4—H4	0.851 (9)
O1—H1B	0.860 (10)	N4—C5	1.395 (2)
N1—C1	1.334 (2)	C1—H1	0.9300
N1—C4	1.334 (2)	C1—C2	1.376 (3)
N2—C3	1.339 (2)	C2—H2	0.9300
N2—C4	1.334 (2)	C2—C3	1.373 (3)
N3—N4^i^	1.423 (2)	C3—H3	0.9300
N3—C5	1.278 (2)	C4—C5	1.480 (2)
			
H1A—O1—H1B	109 (2)	C3—C2—C1	116.54 (16)
C1—N1—C4	115.86 (15)	C3—C2—H2	121.7
C4—N2—C3	116.00 (15)	N2—C3—C2	122.46 (16)
C5—N3—N4^i^	111.21 (13)	N2—C3—H3	118.8
N3^i^—N4—H4	110.2 (15)	C2—C3—H3	118.8
C5—N4—N3^i^	113.47 (13)	N1—C4—C5	117.46 (14)
C5—N4—H4	111.5 (15)	N2—C4—N1	126.25 (15)
N1—C1—H1	118.6	N2—C4—C5	116.30 (14)
N1—C1—C2	122.79 (17)	N3—C5—N4	121.13 (14)
C2—C1—H1	118.6	N3—C5—C4	120.39 (14)
C1—C2—H2	121.7	N4—C5—C4	118.44 (13)
			
N1—C1—C2—C3	−1.6 (3)	N4^i^—N3—C5—C4	178.46 (14)
N1—C4—C5—N3	10.4 (3)	C1—N1—C4—N2	3.1 (3)
N1—C4—C5—N4	−172.09 (16)	C1—N1—C4—C5	−176.87 (15)
N2—C4—C5—N3	−169.62 (15)	C1—C2—C3—N2	1.8 (3)
N2—C4—C5—N4	7.9 (2)	C3—N2—C4—N1	−3.0 (3)
N3^i^—N4—C5—N3	41.7 (2)	C3—N2—C4—C5	176.97 (15)
N3^i^—N4—C5—C4	−135.76 (15)	C4—N1—C1—C2	−0.6 (3)
N4^i^—N3—C5—N4	1.0 (2)	C4—N2—C3—C2	0.4 (3)

Symmetry code: (i) −*x*, *y*, −*z*+3/2.

### Hydrogen-bond geometry (Å, º)

**Table:**

*D*—H···*A*	*D*—H	H···*A*	*D*···*A*	*D*—H···*A*
O1—H1*A*···O1^ii^	0.88 (1)	1.81 (1)	2.642 (4)	156 (2)
O1—H1*B*···N1	0.86 (1)	2.15 (4)	2.863 (3)	140 (5)
N4—H4···N2^iii^	0.85 (1)	2.57 (1)	3.221 (1)	133 (2)
C2—H2···O1^iv^	0.93	2.43	3.278 (3)	151

Symmetry codes: (ii) −*x*+1/2, *y*+1/2, −*z*+3/2; (iii) −*x*, −*y*+1, −*z*+2; (iv) *x*, −*y*, *z*+1/2.
